# Linking health and finance ministries to improve taxes on unhealthy products

**DOI:** 10.2471/BLT.22.288104

**Published:** 2022-06-30

**Authors:** Erika Siu, Anne Marie Thow

**Affiliations:** a Institute for Health Research and Policy, University of Illinois Chicago, 1747 West Roosevelt Road, Chicago, Illinois, 60608, United States of America.; b Menzies Centre for Health Policy and Economics, School of Public Health, Charles Perkins Centre, University of Sydney, Sydney, Australia.

## Abstract

The World Health Organization recommends economic measures such as taxes on tobacco, alcohol and unhealthy foods and beverages as part of a comprehensive strategy for prevention of noncommunicable diseases. However, progress in adopting these so-called health taxes has been hampered, in part, by different approaches and perceptions of key issues in different sectors of government. Health promotion is the responsibility of health policy-makers, while taxation is the mandate of finance ministries. Thus, strengthening cooperation between health and finance policy-makers is central to the successful adoption and implementation of effective health taxes. In this paper we identify the shared concerns of finance and health policy-makers about health taxes with the aim of enabling more effective cross-sector cooperation towards both additional financing for health systems and changes in unhealthy behaviours. For example, new approaches to supporting health taxation include the growing priority for health-system financing due to the growing burden of noncommunicable diseases, and the need to address the health and economic damage due to the coronavirus disease 2019 pandemic. As a result, high-level efforts to achieve progress on health taxes are gaining momentum and represent important progress towards using the combined expertise of health and finance policy-makers.

## Résumé

L'Organisation mondiale de la Santé recommande l'adoption de mesures économiques telles que des taxes sur le tabac, l'alcool ainsi que les boissons et aliments nocifs pour la santé dans le cadre d'une vaste stratégie de prévention des maladies non transmissibles. Cependant, les progrès en la matière ont rencontré des obstacles, notamment en raison de la différence d'approche et de perception des principaux enjeux à divers niveaux du gouvernement. La promotion de la santé relève de la politique sanitaire, tandis que la taxation est la mission du ministère des Finances. Accentuer la coopération entre les responsables de la santé et des finances est donc indispensable à la réussite de l'instauration et de la mise en œuvre de taxes sanitaires efficaces. Dans le présent document, nous identifions les préoccupations partagées tant par les responsables de la santé que par ceux des finances concernant les taxes sanitaires, dans le but d'intensifier la collaboration entre les secteurs. Objectif: débloquer des fonds supplémentaires pour les systèmes de santé et favoriser l'abandon des comportements nuisibles à la santé. Parmi les nouvelles approches de soutien aux taxes sanitaires, citons par exemple une plus grande priorité accordée au financement du système de santé afin de réduire la charge croissante que font peser les maladies non transmissibles, et la nécessité de réparer les dégâts économiques et sanitaires causés par la pandémie de maladie à coronavirus 2019. Ainsi, les efforts visant à développer les taxes sanitaires gagnent du terrain et représentent une avancée considérable vers une valorisation de l'expertise conjointe entre ministère de la Santé et ministère des Finances.

## Resumen

La Organización Mundial de la Salud recomienda la adopción de medidas económicas como los impuestos sobre el tabaco, el alcohol y los alimentos y bebidas poco saludables como parte de una estrategia global de prevención de las enfermedades no transmisibles. Sin embargo, los avances en la adopción de estos llamados impuestos saludables se han retrasado, en parte, por los diferentes enfoques y percepciones de las cuestiones clave en los distintos sectores del gobierno. La promoción de la salud es competencia de los responsables de formular las políticas sanitarias, mientras que la fiscalidad es el mandato de los ministerios de Hacienda. Por lo tanto, el fortalecimiento de la cooperación entre los responsables de formular las políticas sanitarias y financieras es fundamental para el éxito de la adopción y aplicación de sistemas fiscales sanitarios eficaces. En este documento, se identifican las preocupaciones que comparten los responsables de formular las políticas financieras y sanitarias en relación con los impuestos saludables, con el fin de permitir una cooperación intersectorial más eficaz, tanto en lo que respecta a la financiación adicional de los sistemas sanitarios como a la modificación de los comportamientos poco saludables. Por ejemplo, entre los enfoques nuevos para apoyar la fiscalidad sanitaria se encuentran la creciente prioridad de la financiación de los sistemas sanitarios debido a una mayor carga de enfermedades no transmisibles, y la necesidad de solucionar los daños sanitarios y económicos debidos a la pandemia de la enfermedad por coronavirus de 2019. En consecuencia, los esfuerzos de alto nivel para lograr avances en materia de impuestos saludables están cobrando impulso y representan un avance importante hacia el uso de la experiencia combinada de los responsables de formular las políticas sanitarias y financieras.

## ملخص

توصي منظمة الصحة العالمية باتخاذ تدابير اقتصادية مثل فرض ضرائب على التبغ، والكحول، والأطعمة والمشروبات غير الصحية، كجزء من استراتيجية شاملة للوقاية من الأمراض غير المعدية. ومع ذلك، فقد تعرض التقدم في اعتماد هذه الضرائب الصحية للمعوقات بشكل جزئي من خلال الأساليب والمفاهيم المختلفة للقضايا الرئيسية في قطاعات مختلفة بالحكومة. تقع مسؤولية الارتقاء بالصحة على عاتق واضعي السياسة الصحية، في حين أن الضرائب هي من اختصاص وزارات المالية. وبالتالي، فإن تعزيز التعاون بين واضعي سياسات الصحة والمالية يُعد حجر الزاوية لنجاح اعتماد وتنفيذ ضرائب صحية فعالة. نحدد في هذه الورقة الاهتمامات المشتركة لواضعي السياسات المالية والصحية بخصوص الضرائب الصحية التي تهدف لتمكين تعاون أكثر فعالية عبر القطاعات نحو التمويل الإضافي للأنظمة الصحية والتغيرات في السلوكيات غير الصحية. على سبيل المثال، إن الأساليب الجديدة لدعم الضرائب الصحية، تشمل الأولوية المتزايدة لتمويل النظام الصحي بسبب العبء المتزايد للأمراض غير المعدية، والحاجة إلى التعامل مع الضرر الصحي والاقتصادي الناجم عن جائحة مرض فيروس كورونا 2019. ونتيجة لذلك، تكتسب الجهود رفيعة المستوى لتحقيق تقدم في الضرائب الصحية زخمًا، وتمثل تقدمًا مهمًا نحو استخدام الخبرة المشتركة لواضعي السياسات الصحية والمالية.

## 摘要

世界卫生组织建议对烟草、酒精以及不健康的食品和饮料采取经济措施（例如征税），并将其纳入非传染性疾病预防综合战略。但是，因不同政府部门对待关键问题会从不同的角度出发并持有不同看法而导致这些所谓的健康税的采纳进程在一定程度上受到了阻碍。促进健康是健康政策制定者的责任，而税收是财政部的职责。因此，加强健康和财政政策制定者之间的合作对于成功通过和实施有效的健康税至关重要。在本文中，我们发现财政和健康政策制定者对健康税同样关注，以期促进更有效的跨部门合作，从而为卫生系统提供更多资金并改变不健康行为。例如，支持健康税收的新原因包括由于非传染性疾病负担日益加重，卫生系统的筹资问题日益重要，以及需要解决因新型冠状病毒肺炎疫情而造成的健康和经济损失。因此，在各种动力的不断推动下，高级官员正在努力促使健康税取得进展，同时代表着在利用健康和财政政策制定者的综合专业知识方面取得了重要进展。

## Резюме

Всемирная организация здравоохранения рекомендует такие экономические меры, как налоги на табак, алкоголь, нездоровую пищу и напитки, как часть комплексной стратегии профилактики неинфекционных заболеваний. Однако прогрессу в принятии этих так называемых налогов на цели здравоохранения частично препятствовали различные подходы и восприятие ключевых вопросов в различных секторах правительства. За укрепление здоровья отвечают лица, определяющие политику в области здравоохранения, а за налогообложение отвечают министерства финансов. Таким образом, укрепление сотрудничества между лицами, определяющими политику в области здравоохранения, и теми, кто определяет стратегии в области финансов, имеет ключевое значение для успешного принятия и внедрения эффективных налогов на здравоохранение. В этом документе авторы определили общие опасения лиц, определяющих политику в области финансов и здравоохранения, в отношении налогов на здравоохранение с целью обеспечения более эффективного межсекторального сотрудничества как в отношении дополнительного финансирования систем здравоохранения, так и в отношении изменения нездорового образа жизни. Например, новые подходы к поддержке налогообложения здравоохранения включают увеличение приоритета финансирования системы здравоохранения в связи с растущим бременем неинфекционных заболеваний, а также необходимость устранения ущерба для здоровья и экономического ущерба, вызванного пандемией коронавирусной инфекции 2019 года. В результате меры, принимаемые на высоком уровне, по достижению прогресса в отношении налогов на здравоохранение набирают обороты и представляют собой важный прогресс в использовании объединенного опыта лиц, определяющих политику в области здравоохранения и финансов.

## Introduction

Health taxes are those imposed on products that have a negative public health impact. Many countries apply health taxes to products such as tobacco, alcohol and sugar-sweetened beverages that are independent risk factors for noncommunicable diseases. Cardiovascular disease, respiratory disease, cancer and diabetes are estimated to cause 5.64 billion (71%) of the 7.95 billion deaths globally, most of which are premature deaths that occur disproportionately in low- and middle-income countries.^1^ The economic cost of health expenditure and lost productivity is significant,^2^ and households with members having noncommunicable diseases bear a higher risk of impoverishment.^3^

Interventions to prevent noncommunicable diseases are critical to the achievement of sustainable development goal (SDG) 3: to ensure healthy lives and promote well-being for all at all ages. Of particular relevance is target 3.4: by 2030, reduce by one third premature mortality from noncommunicable diseases through prevention and treatment and promote mental health and well-being.^4^ Progress on SDG 3 also plays a key role in the success of socially and economically focused SDGs.

For over 20 years the World Health Organization has endorsed economic measures including taxes in its strategy for prevention of noncommunicable diseases, alongside labelling, marketing restrictions and education initiatives.^1^ There has been growing interest in the use and design of health taxes from organizations such as the United Nations, the International Monetary Fund and the World Bank, suggesting that opportunities exist for collaboration between the health and finance sectors.^5^ In 2021 the United Nations Committee of Experts on International Cooperation in Tax Matters established a Subcommittee on Health Taxes to provide guidance on the implementation of health taxes.^6^

However, the design of health taxes in many countries remains suboptimal, while some countries have yet to adopt such measures, notably sugar-sweetened beverage taxes. One reason for the slow progress has been limited acceptance of the strategy by the finance sector.^7^ The priorities of health and finance policy-makers differ in that finance policy-makers have a mandate to consider the economic interests of industry.^8^ Representatives from the relevant manufacturing industries may claim that high taxation increases illicit trade, pushes consumers to seek cheaper alternatives, and punishes legitimate businesses.^9^ In the United Kingdom of Great Britain and Northern Ireland, arguments against the soft drinks levy from representatives of the sugar-sweetened beverage industry were reminiscent of those used by the tobacco and alcohol industry, and included efforts to undermine public health evidence and the effectiveness of health taxes.^10^ In France, sugar-sweetened beverage taxation was introduced despite legal threats from industry.^11^ Evidence from countries that have successfully introduced health taxes indicates that collaboration between health and finance policy-makers can overcome these challenges and strengthen the design and implementation of health taxes.^12^

We aim to add to the literature by comparing the perspectives of health and finance policy-makers towards health taxes and discussing approaches to building cross-sectoral collaboration in the design and adoption of effective health taxes. 

## Rationale

The first step in health tax policy-making is to justify why health taxes are an appropriate tool to address the concerns of both health and finance policy-makers (Table 1). Consumption of alcohol, tobacco and sugar-sweetened beverages creates a large health and economic burden on both individuals and governments. Country-level estimates can provide evidence of the economic burden of products harmful to health. In Pakistan the combined public and private costs of tobacco-related diseases and deaths in 2019 amounted to an estimated 615.07 billion Pakistani rupees (PKR; equivalent to 3.85 billion United States dollars), mostly due to health-care costs and lost productivity.^13^ This cost is five times the tax revenue collected from the tobacco industry (PKR 120 billion) in the same year.^13^ Thus, these costs ‒ both internal to the user and external to society ‒ are not incorporated into the price of the products.

**Table 1:** Synergies across health and finance policy concerns about health taxes

Key health concerns	Tax policy perspective	Common objectives
*Reduce consumption of health-harming products to reduce the burden of noncommunicable diseases:*	*Reduce negative externalities and internalities through corrective taxes:*	
Health taxes should deter consumers from using products harmful to their health and encourage people towards healthier alternatives	Health taxes should cover the social and personal costs of products harmful to health, without resulting in substitution with other harmful products	Reduce costs to individuals and the health system
*Raise revenue*		
Revenue generated can be used towards subsidizing healthy products or allocated to public health initiatives	Health taxes should generate predictable revenue streams to meet overall government revenue targets	Raise revenue
*Equity impacts*		
Consumers of low socioeconomic status are disproportionately affected by noncommunicable diseases and will experience greater health gains through health taxes	Health taxes should not increase the regressivity of the tax system by creating an unfair burden on lower-income households	Promote equity
*Economic impacts*		
Consumption of harmful products contributes to noncommunicable diseases and decreased individual productivity and participation in the labour force	Health taxes should promote sustainable economic growth and not reduce a country’s overall employment or economic growth	Ensure a healthy workforce and sustainable development that benefits society, economies and the environment
*Tax administration*		
Health taxes should be supported by effective tax administration and not be undermined by illicit trade in harmful products	Health taxes should be transparent and easy to pay and enforce	Prevent illicit trade

Due to the inability of natural market forces to include the full costs of consumption in the price of the product, taxation is necessary as a corrective intervention. This idea was first proposed in 1920 to address externalities, or costs to society,^14^ and later refined by others to include internalities, or information failures of the individual.^15^ Taxes on alcohol, tobacco and sugar-sweetened beverages are considered corrective taxes because they account for personal and social costs by increasing the product price. The higher price is intended to reduce consumption, incentivize industry reformulation of products, generate revenue for government and create a signalling effect of the health risks associated with consumption.^16^ The extent to which an excise tax reduces consumption depends on how demand for a product will be affected by changes in its price ‒ called the price elasticity of demand.^16^ Demand for tobacco and alcohol is price inelastic because consumption declines less than proportionally to the increase in price. In contrast, demand for sugar-sweetened beverages is price elastic, and as price increases, demand decreases.^17^ For similar commodities, such as tobacco and alcohol, taxes on one product have been shown to reduce consumption of both products.^18^

### Maximizing impact 

The appropriate object of a health tax is the product or group of products that are harmful to health. While general sales taxes or value-added taxes are applied across a wide range of goods and services at various stages of the supply chain, a health tax is applied to a specific group of products. The tax drives the price of the target product higher relative to other goods, creating a disincentive for consumption. The object of a health tax is best defined through collaboration between a government’s health and finance ministries in the process of tax design, making use of local and global health evidence and policy recommendations.^8^

The tax base and tax rate directly affect the price of a product and the revenue generated. The tax base is the value, quantity or volume of a product or ingredient on which a tax rate is applied. Taxes based on value are known as *ad valorem* taxes, where the tax rate is applied to the value of the product at some point along the value chain. For example, in 2016, the Gulf Cooperation Council countries agreed to adopt a 100% uniform *ad valorem* tax on the retail price of all tobacco products.^19^ A more common alternative is to adopt specific or *ad rem* taxes, which are based on a defined unit or volume of a product or the measure of a key ingredient: for example, the French sugar-sweetened beverage tax of 0.0716 euro per L.^11^ Over the last decade many countries have moved away from *ad valorem* taxes to adopt specific or mixed tax systems.^20^

Health tax rates should increase over time to keep pace with a country’s economic growth and to further curb consumption. Research has shown that significant increases in tax rates on cigarettes work to raise prices and result in sustained decreases in the prevalence of smoking, while acting as a deterrent to new smokers.^21^ In Ukraine, the government increased the average excise tax rate on cigarettes 10-fold between 2008 and 2015, which increased the price by 400%.^21^ As a result, smoking prevalence decreased by nearly one third in the same period,^21^ with expected long-term health gains and interruption of habit formation. Taxes on sugar-sweetened beverages are likewise correlated with reductions in calorie intake due to these drinks.^22^ Ultimately, the choice of tax rate should depend on the health goal of reducing consumption and the revenue target, both of which are informed by consumer responses to the price increase, or the price elasticity of demand. Evidence suggests that tax measures perceived as having advantages for both revenue-raising and health objectives acquire greater support across government ministries than those with an exclusive objective.^8^

Strategic design of health taxes can mitigate the potential for consumers substituting products to undermine the effectiveness of taxes. There is evidence of substitution from taxed cigarettes to lesser or untaxed tobacco products, such as electronic cigarettes, suggesting that all tobacco products should be taxed at similar levels.^23^ On the other hand, health taxes can encourage substitution with healthier alternatives, and provide incentives to manufacturers to reduce the sugar or alcohol content of their products. Alcohol and sugar-sweetened beverage taxes often use differential rates to deter consumers from the most harmful products, while encouraging them towards the least harmful products. An example is the United Kingdom soft drinks industry levy, which includes two tiers: 0.10 pounds sterling (£) per L for drinks containing between 5 and 8 grams of sugar per 100 mL and £0.24 per L for drinks containing more than 8 grams of sugar per 100 mL.^24^

## Revenue impacts

For finance policy-makers, health taxes must be considered within a system of general government and health budgeting targets (Table 1).^5^ Excise taxes may be perceived as insignificant or unreliable revenue streams, due to the limited amount of potential tax revenue relative to other taxes, such as general sales taxes and the ability to deter consumer purchases. Another concern for finance policy-makers is a tipping-point in revenues when the gain due to higher taxes starts to be reduced as consumption decreases. Indeed, revenues often decline in countries without laws against frontloading of production. Frontloading involves over-producing a product before a tax increase and then under-producing it after the tax increase, thus lowering government revenues.^25^ Industry representatives can then attribute this revenue loss to such a tipping-point. Although the aim of health taxes is to decrease the public’s consumption of specific products, inelastic demand for products such as alcohol and tobacco means that revenue streams from these taxes are relatively reliable in the long term, and no country has yet reached such a tipping-point.^26^

In the context of public health and economic recovery from the coronavirus disease 2019 (COVID-19) pandemic, governments worldwide are seeking additional sources of revenue. The opportunity to raise revenue without raising income taxes on earnings can be attractive to a government, particularly during economic crises. Following the global financial crisis of 2008–2010, several European countries adopted sugar-sweetened beverage taxes as part of their recovery package.^12^

### Maximizing revenue impact

Uniform specific taxes are generally recommended for the taxation of harmful products because they are transparent, easy to administer and less susceptible to price manipulation, as they are assessed on the unit, dose or volume of a product, rather than its value. For this reason, specific taxes provide predictable streams of government revenue. Mixed tax structures are comprised of both specific and *ad valorem* components, or an *ad valorem* tax with a minimum tax floor. The specific or minimum tax component reduces the price gap between brands and discourages consumers from substituting lower-priced brands for their usual product, so that the government’s health objectives can be achieved. Meanwhile the *ad valorem* component allows governments to gain more revenue from higher-value products.^27^ However, the greater complexity of mixed structures increases the challenges of tax administration.

If the rationale for the tax is accepted as directed towards public health, there is a further question of whether the tax revenues should be earmarked for the health system. Some public finance experts argue that earmarked tax revenues on harmful products may reduce the allocation for health in the general budget, while creating rigidity for governments in the allocation of public funds.^28^^,^^29^ However, at least 33 countries already earmark health taxes for health promotion purposes,^30^ and tax increases intended for specific health purposes have been found to receive greater public support. For example, in the Philippines alcohol and tobacco taxes were earmarked for a universal health coverage scheme in line with politicians’ election promises.^31^ Many countries informally earmark tax revenue for social benefits and public health. For example, in France 50% of sugar-sweetened beverage tax revenue is earmarked for the social security system.^11^

## Equity impacts

Equity is important to both health and finance policy-makers, and health taxes can be designed in a way to promote both health and economic equity (Table 1). Health taxes often have a larger impact on consumption in population subgroups that are less responsive to other interventions, such as youth, poorer people and pregnant women.^32^^–^^34^ Lower socioeconomic groups are relatively more responsive to tobacco price changes than higher socioeconomic groups.^35^^,^^36^ In the United States of America an analysis of population responses to a state-level tax found that individuals of low socioeconomic status were almost twice as likely as those of higher socioeconomic status to report reductions in smoking.^37^ Another study of six countries in south-east Europe found that lower-income households in all countries were more responsive to price increases than high-income households, and in some of the countries the share of the household budget dedicated to cigarettes decreased, even as cigarette prices rose.^38^

Global evidence shows that reduced consumption of products harmful to health is significantly associated with reduced health-care costs and increased productivity.^39^ For example in Mexico, researchers estimated that a 58% increase in the price of cigarettes would lead to a 4% increase of available income for low-income households, through a combination of medical expenses saved and additional years of employment due to prevention of smoking-attributable disease and premature death.^40^

### Maximizing equity impact

The design of a health tax should therefore consider the price elasticities of demand among socioeconomic groups. Medium- to long-term impacts, such as medical expenses avoided and increased productivity, should also be recognized.

The adoption of command and control measures in addition to taxation can enhance the capacity of low-income consumers to reduce their consumption of harmful products.^41^ For example, services to help consumers cease use of harmful products, along with measures such as smoke-free areas and graphic health warning labels, can further support individuals’ reduction of tobacco consumption.

Using the tax revenues to provide services to low-income populations promotes equity.^42^ The use of revenue (whether formally or informally earmarked) can contribute to wealth redistribution and mitigate health inequalities.^43^ For example, the soft drinks industry levy in the United Kingdom was informally committed to new expenditure on school-based health-promotion programmes.^10^

## Economic impacts

The predominant questions of finance policy-makers centre on the broader macroeconomic impact of health taxes (Table 1). How would a tax affect employment in the affected manufacturing sector, related economic sectors and the overall economic growth of the country? Finance policy-makers are confronted with industry opposition to health taxes on these grounds.^10^ Industry representatives commonly point to the potential negative impact of health taxes due to job losses for farmers and industry workers. However, there is empirical evidence that many more jobs can be created in more beneficial sectors by taxing tobacco and using the revenues in other sectors. In Pakistan for example, researchers simulated an increase of the effective excise tax share on tobacco to 70% and found that although the tax increase would result in a loss of 13 150 jobs in the cigarette industry, a net increase of 308 550 new jobs would be created economy-wide through shifts of household and government spending to other sectors.^44^

### Economic considerations

It is important that both health and finance policy-makers take the broader economic impacts of the health tax into consideration when designing health taxes. Understanding the size of the relevant manufacturing sector in relation to a country’s overall economy is an important first step. Implementing programmes to help workers to transition to other livelihoods will also alleviate negative impacts. For example, the sin tax reform law enacted in the Philippines in 2012 allocated 15% of the tobacco tax revenues to local governments for cash transfers to support farmers’ livelihoods, and the remaining revenues were allocated to universal health coverage for older and low-income people.^45^

## Tax administration

Finally, health and finance policy-makers are concerned with tax administration (Table 1). Illicit trade can undermine both the health and revenue goals of a tax and may occur through informal markets, a lack of regulation or enforcement, a lack of coordination within and between governments or the presence of corruption.

Licensing and monitoring of industry production and sales, along with systematic and continuous independent assessment of illicit trade in harmful products, is paramount to effective administration of health taxes. Self-reporting by industry has shown to be ineffective, as it results in underreporting of production and consequent loss of government revenue.^46^ Enforcement of increases in alcohol and tobacco taxes is more effective when preceded by the strengthening of anti-smuggling laws and border controls.^47^^,^^48^ Such efforts require government ministries to coordinate with law enforcement and border and customs agencies to exchange information and combine operations.^47^^,^^49^ Finally, coordination between countries is essential for preventing entry of illicit products across national borders.^20^

### Administration considerations

Effective administration involves ensuring compliance with health taxes through making the taxes as transparent and simple as possible to pay and collect, monitoring industry production, sales and tax revenues, and enforcing the law through seizures of illicit products and assessment of penalties as provided by law.

A simple and transparent tax design increases manufacturers’ and distributors’ compliance and reduces administration costs and opportunities for tax avoidance and evasion. Uniform specific taxes are based on the quantity, volume or dose of a product ‒ not the value of the product, which is subject to manipulation ‒ and are thus easier to administer relative to *ad valorem* taxes. Complex tax structures create more opportunities for manufacturers to avoid tax, including where the tax base of an *ad valorem* system is not the final retail price. Finally, taxing earlier in the supply chain of the harmful products reduces the number of taxable entities, or subjects, and makes them easier to identify and monitor.^20^

## Conclusion

We have described the key policy concerns of finance and health policy-makers in relation to health taxes and provided policy recommendations for the design and implementation of health taxes (Box 1). With a clear understanding of shared objectives, mutual concerns and existing evidence, policy-makers can bridge the gap between the health and finance sectors to achieve the desired outcomes for both health and revenue. After the health and economic damage caused by the COVID-19 pandemic, health taxes offer an opportunity for governments to address revenue and health priorities. Strengthening understanding between the health and finance sectors can take advantage of the combined expertise of health and finance policy-makers to build agreement towards mobilization of domestic resources and public health promotion in line with the sustainable development agenda.

Box 1 **Summary of key policy design recommendations for health taxes**

**Revenue impacts**

- Apply uniform specific health taxes (without tax tiers across brands or prices) or mixed tax structures (both specific and *ad valorem* taxes)
- Earmark health taxes to public health spending to win public acceptance

**Equity impacts**

- Adopt other tobacco control measures, such as graphic warning labels on products or services to help consumers cease use of harmful products
- Use revenues from health taxes to fund services for low-income groups

**Economic impacts**

- Understand the generally small size of industries producing harmful products in relation to the whole economy
- Implement programmes to help workers in industries that produce harmful products to transition to other livelihoods

**Tax administration**

- Simplify tax design so that health taxes are easy to pay and collect
- License and monitor industry production and sales of harmful products
- Verify industry production reporting on volumes of harmful products
- Tax harmful products early in the supply chain at the producer level
